# Where are the children of working parents during school closures? Sigrid Mairhofer

**DOI:** 10.1093/eurpub/ckac131.097

**Published:** 2022-10-25

**Authors:** S Mairhofer, B Plagg, H Flarer

**Affiliations:** 1 Applied Social Sciences, Hochschule München University of Applied Sciences, Munich, Germany; 2 Institute of General Practice and Public Health, Provincial College for Health Professions Claudiana, Bolzano, Italy; 3 Faculty of Education, Free University of Bozen - Bolzano, Bolzano, Italy; 4 Eurac, European Research Academy, Bolzano, Italy

## Abstract

Italy was the first European country to be hit hard by the pandemic and in the context of the Covid-19 pandemic, there were several closures of education and care facilities. The first closure of education facilities lasted continuously from March to September 2020, followed by numerous shorter closures. However, if working parents continue to (have to) work, the question arises who will look after the children during this time when facilities close. To answer this question a quantitative survey of working parents during a ‘hard closure’ week in February/March 2021 was conducted in the province of Bolzano (Northern Italy). 3725 adults as parents of a total of 7,372 children from different households responded. Although not officially allowed, 53.4% of all participants sought help from people outside the nuclear family to bridge the situation, mostly from grandparents (79%; n = 1855). The situation that grandparents represented the main risk group at the time and could not yet have sufficient vaccination protection appears particularly worrying here. Other parents’ coping strategies included working early in the morning or at night (23 %; n = 850), or leaving children unsupervised (25 %, n = 929).

**Conclusions:**

School closures shift families to new strategies, including unhealthy models of alternating work/childcare, ‘illegal’ involvement of third parties outside the nuclear family, and neglect of age-appropriate childcare. Our findings highlight that when childcare facilities are repeatedly closed, working families need additional support strategies to reduce contact and minimize secondary harms.

**Key messages:**

• When schools close, parents have to find new strategies for where to take their children, many have contacts outside the home.

• Grandparents (also as a risk group) play a major role in childcare even during a lockdown.

